# Assessment of prevalence and risk factors associated with Hepatitis B virus infection among blood donors in Mogadishu Somalia

**DOI:** 10.1186/s12889-024-18136-2

**Published:** 2024-03-04

**Authors:** Abdirahman Khalif Mohamud, Pamornsri Inchon, Sirinan Suwannaporn, Kriengkrai Prasert, Najib Isse Dirie

**Affiliations:** 1 Faculty of Medicine and Health Sciences, SIMAD University, Mogadishu, Somalia; 2 Department of Public Health, School of Health Science, Mae Fah Luang University, ChiangRai, Thailand; 3 Nakhon Phanom Provincial Hospital, Nakhon Phanom, Thailand; 4 Faculty of Public Health, Kasetsart University, Sakon Nakhon, Thailand; 5 Department of Urology, Faculty of Medicine, and Health Sciences, Dr Sumait Hospital, SIMAD University, Mogadishu, Somalia

**Keywords:** Prevalence, Factors associated with HBV infection, Hepatitis B vaccine, Mogadishu, Somalia

## Abstract

**Background:**

The Hepatitis B virus (HBV) is transmitted through contaminated blood or bodily fluids. Globally, over 81 million blood units are donated annually, a crucial therapeutic procedure without alternatives. However, blood-borne infections, including HBV, pose a significant hurdle to safe transfusions, especially in HBV-endemic regions like Somalia with limited screening. Therefore, this study aims to estimate the prevalence of Hepatitis B virus infection and identify risk factors associated with it among blood donors in Mogadishu, Somalia.

**Method:**

A hospital-based cross-sectional study was conducted between February and April 2023. Research tools included a 5-ml blood sample and a structured questionnaire. The presence or absence of HB markers was determined using a multi-HB rapid test and CDC’s HB marker interpretation guideline. Logistic regression was used in univariate and multivariate models to identify risk factors associated with HBV infection, with significance set at a p-value < 0.05 in the final model.

**Result:**

A total of 494 blood donors were recruited for this study; 93.9% were male, with a mean age of 31.5 (SD = 8.11). The prevalence of Hepatitis B virus (HBV) infection among blood donors was 9.7%, with a 95% CI of 7.1–12.3. In multivariable logistic regression, those with a monthly income of less than 200 USD (AOR = 5.20, 95% CI = 1.61–16.79), those with an income between 200 and 400 (AOR = 3.59, 95% CI = 1.38–9.34), Jobless blood donors (AOR = 3.78, 95% CI = 1.17–12.20), those in business occupations (AOR = 3.35, 95% CI = 1.24–9.08), those with a history of STDs (AOR = 4.83, 95% CI = 2.03–11.50), those without a history of HB vaccine (AOR = 13.81, 95% CI = 2.46–77.41), those with a history of tooth extraction (AOR = 6.90, 95% CI = 2.66–17.88), and those who shared sharp equipment (AOR = 2.90, 95% CI = 1.07–7.82) were more likely to become infected with the Hepatitis B virus (HBV) compared to their counterparts.

**Conclusion:**

This study highlights a high prevalence of Hepatitis B virus (HBV) infection. Implementation efforts against HBV infection should specifically focus on low-income individuals, the jobless, and donors with a history of STD to mitigate the burden of HBV infection and promote safer blood donation. In addition, discouraging the sharing of sharp equipment, improving infection control practices during tooth extraction procedures, and enhancing HB vaccination uptake, particularly among individuals lacking a history of HB vaccine, is highly recommended.

## Background

The Hepatitis B virus (HBV) belongs to the Hepadnavirus family, causing hepatitis and transmitting through contaminated blood or other bodily fluids [1]. HBV poses a life-threatening liver disease, often remaining asymptomatic, and carriers may unknowingly spread it [2]. Globally, approximately 240 million people live with chronic HBV infection, resulting in over 300 thousand complications related to liver diseases such as damage, cirrhosis, or liver cancer, contributing to 68,600 deaths [3, 4]. The prevalence of HBV exceeding 8% is categorized as high, while 2 to 7% is considered medium, and less than 2% is labeled as low endemicity [4, 5].

Globally, more than 81 million blood units are donated each year, and blood transfusion stands as a crucial therapeutic procedure without a viable alternative, essential for saving millions of individuals requiring transfusions [4–6]. However, blood-borne infections, such as HBV, present a significant hurdle to ensuring safe transfusions, particularly in HBV-endemic regions where effective screening methods are limited [5]. The transfusion of contaminated blood contributes to as many as 16 million new HBV infections worldwide, with each blood unit carrying a 1.0% risk of transmitting blood-borne infections [6, 7]. Moreover, unsafe blood transfusions are projected to lead to approximately 45,000 new HBV infections in Africa alone, and about 1.6 million blood units are discarded annually due to blood-borne infections, including HBV [1]. As a result, 12.5% of transfusion recipients in Africa are at risk of contracting hepatitis following transfusions [8].

Hepatitis B Virus has remained a neglected concern in Africa, with over 60 million Africans infected [8]. Among African blood donors, the prevalence of HBV ranges from 5 to 7% [1]. In different regions of Africa, the prevalence of HBV among blood donors varies it’s 18.60% in Southwest Nigeria, 4.10% in Calabar Nigeria [9, 10], 5.60% in Kenya [11], 10.90% in Jijiga Ethiopia [12], and 2.0% in Eritrea [13]. The primary HBV risk factors include having unprotected multiple sexual partners, sharing injected needles or syringes, using unsterilized medical equipment, and exposure to contaminated objects such as poorly sterilized medical, surgical, and dental equipment [1, 6, 7, 9, 11, 14].

Somalia confronts a shortage of data concerning HBV infection among blood donors, particularly in Mogadishu, its capital and most densely populated city. Clinical facilities in Somalia contend with a substantial demand for blood transfusions, coupled with a high prevalence of HBV infection, attributed to factors such as frequent disaster incidents, road traffic accidents, endemic malaria, childhood anemia, and blood loss from surgical and obstetric procedures [15–19]. A 19.0 and 2.1% HBV prevalence among Somali blood donors were reported in 1995 and 2023 respectively [15, 16]. A meta-analysis disclosed an 18.9% pooled HBV prevalence among the general population in Somalia and 7.3% of HBV prevalence among hemodialysis patients were also reported [17, 18]. Although studies excise an investigation into associated risk factors has been lacking. Acquiring current information about the associated risk factors in Somalia is crucial to ensure a safe blood supply and reduce the risk of spreading blood-borne infections, particularly HBV. This information is vital for both preventing and treating HBV, supporting national and international HBV prevention strategies, and eradicating programs. Hence, this study aims to estimate the prevalence of Hepatitis B virus infection and identify risk factors associated with blood donors in Mogadishu, Somalia.

## Method

### Study design

A hospital-based cross-sectional study was conducted between February and April 2023 at Banadir Public Hospital in Mogadishu, Somalia.

### Study setting

The study was carried out at Banadir Hospital, situated in Mogadishu, the capital, and a densely populated city. It stands as one of the largest teaching and referral hospitals and is one of the two public hospitals in the capital city run by the Somali Government. Since its establishment in 1976, the hospital has offered medical and surgical services to over 3 million patients. With more than 700 beds and a staff of 400, the hospital admits between 2,500 and 3,000 patients monthly. Due to its high admission rates and it conducts blood donation campaigns to meet the demand for blood transfusions the substantial number of blood donors from across the city makes it an ideal choice as the study hospital [20, 21].

### Study population

The study population was all blood donors attending the Banadir Hospital’s blood bank during the data collection period, providing five-milliliter blood samples for screening and consenting to participate in the study.

### Inclusion and exclusion criteria

The study included all blood donors attending the blood bank of the study hospital during the data collection period to donate blood or blood components. Exclusions were made for those not mentally fit, unwilling to provide consent or a blood sample, unable to communicate verbally or hear, and those who had received a dose of the hepatitis B vaccine within the last 30 days. The Centers for Disease Control and Prevention (CDC) suggested that individuals who received the HB vaccine within the past 30 days might transiently exhibit HBsAg positivity without infection [22].

### Sample size and sampling technique

A standardized formula for cross-sectional studies was utilized to calculate the required sample size [23]. The formula used was n = Z^2^_α/2_ *P*(1 - P) /d^2^: Where Z^2^_α/2_ is 1.96, P represents the proportion of prevalence, determined to be 18.6% based on a similar study conducted in Nigeria [14], and d is the desired precision level set at 0.05. Consequently, this study necessitated a minimum sample of 256 participants, with an additional 10% accounting for potential non-response. However, the study had the necessary resources to accommodate a sample size of up to 494, exceeding the minimum requirement of 256. This larger sample size ensures a representative subset of the population, contributing to accurate outcome inference, enhanced representativeness, increased precision of estimates, improved generalizability of findings, and facilitation of more robust subgroup analyses [23–25]. Meanwhile, the reported prevalence of HBV infection in African countries between 1995 and 2023 has varied, ranging from 2.1 to 19.0% [2, 9, 12, 15, 17, 18]. In addition, a systematic sampling technique was used, involving the selection of blood donors one and five in every 10 blood donors.

### Research instrument

A 5 mL blood sample and a well-structured, reliable, validated questionnaire were developed from a literature review and used as research tools. The questionnaire was initially developed in English and was verbally translated during the interviews for data collection. The Item Objective Congruence (IOC) technique, performed by three external field experts (an infectious disease specialist, a public health researcher, and a clinical epidemiologist), was used to validate the questionnaire [26]. Subsequently, a pilot study involving 30 respondents with similar characteristics was conducted at the study hospital, yielding an acceptable Cronbach’s alpha of 0.76. The CDC’s standardized HB serological interpretation guideline was utilized to interpret HB markers [22].

The questionnaire comprises four sections: **(i)** Socio-demographic characteristics, including blood donors’ age, sex, marital status, level of education, occupation, monthly income, and residential area. **(ii)** Sexual, behavioral, and blood donation characteristics, including the number of previous marriages and HBV screening before marriage if ever married, history of STDs, cigarette smoking, chewing khat, and condom use, history of receiving blood, frequency and type of donation, history of HB vaccination, and doses of HB vaccine received. **iii)** Risk characteristics, such as the history of sharing injected needles, history of sharing sharp equipment, history of tooth extraction, history of sharing tissue transplantation, history of haemodialysis, history of surgical operations, history of hospitalizations, history of sharing accidental needle injuries, e*nsuring barber changing the blade, and* having HBV-infected family members. Individuals who had previously experienced STDs such as syphilis, herpes, human papillomavirus (HPV), chancroid, gonorrhea, and chlamydia within the last two years were identified as having a history of STDs [27].

### Laboratory and outcome definitions

A rapid, qualitative immunoassay (Advanced Quality One-Step Multi-HBV test) was employed to determine the presence or absence of HBV markers in human blood, serum, or plasma. The sensitivity and specificity of HBV markers were as follows: HBsAg (99.85% sensitivity and 99.90% specificity), HBsAb (99.70% sensitivity and 99.41% specificity), HBeAg (100% sensitivity and specificity), HBeAb (99.34% sensitivity and 90.93% specificity), and HBcAb (99.78% sensitivity and 99.74% specificity) [28]. The Centers for Disease Control and Prevention (CDC) standardized HB serological interpretation guideline was utilized for interpreting HB markers [22]. Any blood sample showing positive HBsAg and/or anti-HBs was considered to have HBV infection, after excluding those with anti-HBc negative, indicating an absence of HBV exposure and no evidence of recent, past, resolved, or chronic HBV infection [22].

### Data collection procedure

Three licensed medical professionals collected data after undergoing five days of training to enhance their understanding of the questionnaire content and blood sample collection procedures. They assessed blood donors’ eligibility for study participation, informed eligible donors about the study, and requested their participation. Agreed participants signed a consent form or provided a fingerprint for illiterate donors. Subsequently, a 5 mL blood sample was collected, and a face-to-face interview was conducted with each participant, following the research instrument, lasting approximately 20 min individually.

### Data analysis procedure

The collected data were cleaned, coded, and entered into a spreadsheet, then imported into SPSS version 20 (SPSS, Chicago, IL License) for analysis. Descriptive statistics were used by presenting frequencies with percentages for all categorical characteristics and means with standard deviations (SD) for continuous variables. The overall prevalence of HBV infection was presented as frequency with percentage and its 95% CI. Logistic regression in univariable and multivariable models was used to determine factors associated with HBV infection. Variables with a p-value < 0.20 in the univariable model were selected as candidates for inclusion in the multivariable model. Bursac et al. [29] suggested that a p-value < 0.20 for univariable logistic regression might indicate some reasonable association with the outcome in the final model due to the possibility of confounding variables. In the multivariable logistic regression model, the Hosmer-Lemeshow goodness-of-fit test was used to assess the final model’s goodness of fit [30]. Variables with a p-value < 0.05 were considered statistically significant.

### Result

The prevalence of hepatitis B virus (HBV) infection among blood donors was 9.7% with a 95% confidence interval of 7.1–12.3 (Table 1).

**Table 1:** Prevalence of Hepatitis B virus infection among blood donors

Characteristics	n	%	95%CI
Prevalence of hepatitis B virus (HBV)			
**Infected**	48	9.7	7.1–12.3
Non-infected	446	90.3	87.7–92.9

Most of the study participants (93.9%) were male, with a mean age of 31.58 (SD = 8.11). Over half (57.1%) were single, and 30.5% were illiterate. Most participants (42.5%) were employed, 38.9% had a monthly income higher than 400 USD, and 80.6% were urban residents (Table 2).

**Table 2:** Socio-demographical characteristics between HBV-infected and non-infected

Characteristics	HBV infection		Total n (%)	p-value
	Infected n (%)	None Infected n (%)		
**Sex**				
Male	46 (95.8%)	418 (93.7%)	464 (93.9%)	0.757^a^
Female	2 (4.2%)	28 (6.3%)	30 (6.1%)	
**Age**				
18–28	17 (35.4%)	161 (36.1%)	178 (36.0%)	0.887
29–39	25 (52.1%)	219 (49.1%)	244 (49.4%)	
≥40	6 (12.5%)	66 (14.8%)	72 (14.6%)	
Min = 18, Max = 60, Mean = 31.58, SD = 8.116				
**Marital status**				
Single	29 (60.4%)	253 (56.7%)	282 (57.0%)	0.976^a^
Married	14 (29.2%)	134 (30.0%)	148 (30.0%)	
Window	3 (6.2%)	37 (8.3%)	40 (8.1%)	
Divorced	2 (4.2%)	22 (4.9%)	24 (4.9%)	
**Level of Education**				
Illiterate	16 (33.3%)	135 (30.3%)	151 (30.6%)	0.664
Read or write	15 (31.1%)	114 (25.6%)	129 (26.0%)	
Primary level	11 (22.9%)	137 (30.7%)	148 (30.0%)	
Post-primary	6 (12.5%)	60 (13.5%)	66 (13.4%)	
**Occupation**				
Employee	19 (39.6%)	191 (42.8%)	210 (42.5%)	0.068
Business	12 (25.0%)	118 (26.5%)	130 (26.3%)	
Healthcare	3 (6.2%)	67 (15.0%)	70 (14.2%)	
Jobless	14 (29.2%)	70 (15.7%)	84 (17.0%)	
**Residential area**				
Rural	7 (14.6%)	89 (20.0%)	96 (19.4%)	0.371
Urban	41 (85.4.3%)	357 (80.0%)	398(80.6%)	
**Monthly Income USD** $				
<200	18 (37.5%)	112 (25.1%)	130 (26.3%)	0.072
200–400	18 (37.5%)	154 (34.5%)	172 (34.8%)	
>400	12 (25.0%)	180 (40.4.8%)	192 (38.9%)	
Min = 0, Max = 3000, Mean = 440.58, SD = 335.80				

^*^Significant level at a p-value < 0.05

^a^ Fisher’s exact test

This study revealed that 39.2% of ever-married respondents had previously married three times. over half (55.2%) of them did not screen for HBV infection before marriage. Slightly less than two-thirds (61.7%) did not use a condom, 83.6% did not have a history of sexually transmitted diseases (STDs), 78.1% did not smoke cigarettes, and 83.6% did not chew khat. In addition, 58.9% of the participants were first-time blood donors, 61.9% were voluntary donors, and more than two-thirds (76.9%) had a history of blood receiving. Only 15% of the study participants had previously received the hepatitis B vaccine, and almost half of them (55.4%) had only one dose of HB vaccination (Table 3).

**Table 3:** Behavioral, sexual, and blood donation characteristics between HBV-infected and non-infected

Characteristics	HBV infection		Total n (%)	p-value
	Infected n (%)	None Infected n (%)		
**If ever married, the number of previous marriages**				
1 Marriage	2 (10.5%)	31 (16.1%)	33 (15.6%)	0.213^a^
2 Marriages	5 (26.3%)	61 (31.6%)	66 (31.0%)	
3 Marriages	6 (31.6%)	77 (39.9%)	83 (39.2%)	
≥4 Marriages	6 (31.6%)	24 (12.4%)	30 (14.2%)	
**If ever married, Screen HBV before marriage**				
We do both	2 (10.5%)	29 (15.0%)	31 (14.6%)	0.075^a^
One of us do	2 (10.5%)	47 (24.4%)	49 (23.1%)	
We do not do both	11 (57.9%)	106 (54.9%)	117 (55.2%)	
Not sure/do not remember	4 (21.1%)	11 (5.7%)	15 (7.1%)	
**Condom use**				
Yes (Every time)	4 (8.3%)	60 (13.5%)	64 (13.0%)	0.319
Yes (Sometimes)	5 (10.4%)	34 (7.6%)	39 (7.9%)	
No	34 (70.8%)	271 (60.8%)	305 (61.7%)	
I do not do sex	5 (10.4%)	81 (18.2%)	86 (17.4%)	
**History of STD**				
Yes	26 (54.2%)	55 (12.3%)	81 (16.4%)	< 0.001*
No	22 (45.8%)	391 (87.7%)	413 (83.6%)	
**Cigarette smoking**				
Yes	20 (41.7%)	88 (19.7%)	108 (21.9%)	< 0.001*
No	28 (54.3%)	358 (80.3%)	389 (78.1%)	
**Chewing khat**				
Yes	16 (33.3%)	65 (14.6%)	81 (16.4%)	0.001*
No	32 (66.7%)	381 (85.4%)	413 (83.6%)	
**Frequency of blood donation**				
First time donner	37 (77.1%)	254 (57.0%)	291 (58.9%)	0.007*
Repeated donner	11 (22.9%)	192 (43.0%)	203 (41.1%)	
**Type of blood donate**				
Relative donation	20 (41.7%)	168 (37.7%)	188 (38.1%)	0.588
Volunteer donors	28 (58.3%)	276 (61.9%)	306 (61.9%)	
**History of receiving blood**				
Yes	20 (41.7%)	94 (21.1%)	114 (23.1%)	0.001*
No	28 (58.3%)	352 (78.9%)	380 (76.9%)	
**History of HB vaccination**				
No	46 (95.8%)	374 (83.9%)	420 (85%)	0.027*
Yes	2 (4.2%)	72 (16.1%)	74 (15%)	
**Doses of HB vaccine received**				
One dose	0 (0.0%)	41 (56.9.0%)	41 (55.4%)	0.017*^a^
Two doses	0 (0.0%)	23 (31.9%)	23 (31.1%)	
Three doses	2 (100.0%)	8 (11.1%)	10 (13.5%)	

^*^Significant level at a p-value < 0.05

^a^ Fisher’s exact test

The study showed that 11.7% of the participants had a history of sharing injected needles, 43.9% of their barbers did not change the blade, 23.3% shared sharp equipment, 20.0% had a history of tooth extraction in the last two years, 21.7% had a history of tissue transplantation, 24.7% had a history of toothbrush (Miswak) or razor sharing, 0.4% had a history of hemodialysis, 20.6% had a history of surgical operation, 22.1% had a history of hospitalization, 17.0% had an HBV infected family member, and 17.6% had a history of accidental needle stick injury (Table 4).

**Table 4:** Risk characteristics between HBV-infected and non-infected

Characteristics	HBV infection		Total n (%)	p-value
	Infected n (%)	Non-infected n (%)		
**History Sharing injected needles**				
Yes	14(29.2%)	44(9.9%)	58(11.7%)	< 0.001*
No	34(70.8%)	402(90.1%)	436(88.3%)	
**Ensuring the barber changing the blade**				
Yes (Every time)	16(33.3%)	148(33.2%)	164(33.2%)	0.002*
Yes (Sometimes)	20(41.7%)	93(20.9%)	113(22.9%)	
No	12(25.0%)	205(46.0%)	217(43.9%)	
**History Sharing sharp equipment**				
Yes	33(68.8%)	82(18.4%)	115(23.3%)	< 0.001*
No	15(31.2%)	364(81.6%)	379(76.7%)	
**History of tooth extraction**				
Yes	33(68.8%)	66(14.8%)	99(20.0%)	< 0.001*
No	15(31.2%)	380(85.2%)	395(80.0%)	
**History of tissue transplantation**				
Yes	19(39.6%)	88(19.7%)	107(21.7%)	0.002*
No	29(60.4%)	358(80.3%)	387(78.3%)	
**History of hemodialysis**				
Yes	1(2.1%)	1(0.2%)	2(0.4%)	0.185
No	47(97.9%)	445(97.9%)	492(99.6%)	
**History of surgical operation**				
Yes	19 (39.6%)	83 (18.6%)	102 (20.6%)	0.001*
No	29 (60.4%)	363 (81.4%)	392 (79.4%)	
**History of hospitalized**				
Yes	21 (43.8%)	88 (19.7%)	109 (22.1%)	0.001*
No	27 (56.2%)	358 (80.3%)	385 (77.9%)	
**HBV-infected family member**				
Yes	13 (271.1%)	71 (15.9%)	84 (17.0%)	0.118
No	31 (64.6%)	345 (77.4%)	376 (76.1%)	
I do not know	4 (8.3%)	30 (6.7%)	34 (6.9%)	
**History of sharing accidental needle injury**				
Yes	20 (41.7%)	67 (15.0%)	87 (17.6%)	< 0.001*
No	28 (58.3%)	379 (85.0%)	407 (82.4%)	

^*^Significant level at a p-value < 0.05

^a^ Fisher’s exact test

In univariable logistic regression analysis, seventeen (17) variables were found to be significantly associated with HBV infection at a p-value < 0.20. These variables included respondents’ occupation, income, history of STD, cigarette smoking, chewing khat, frequency of blood donation, history of blood receiving, history of HB vaccine, history of sharing injected needles, history of sharing sharp equipment, ensuring barber changing the blade, history of tooth extraction, history of tissue or organ transplantation, history of hemodialysis, history of surgical operation, history of hospitalization, and history of accidental needle stick injuries. These seventeen variables were candidates in multivariable logistic regression, and six were found to be significantly associated with HBV infection among blood donors at a p-value < 0.05.

Those with a monthly income of < 200 USD had 5.20 times greater risk (95% CI = 1.61–16.79), those with income between 200 and 400 had 3.59 times (95% CI = 1.38–9.34) greater risk of HBV infection compared to those with income > 400 USD. Jobless blood donors had a 3.78 times greater risk (95% CI = 1.17–12.20), while those in business occupations had a 3.35 times greater risk (95% CI = 1.24–9.08) of HBV infection compared to employees. Those with a history of STD were 4.83 times (95% CI = 2.03–11.50) more likely to be HBV infected compared to those without. The odds of HBV infection were 13.81 (95% CI = 2.46–77.41) for those without a history of HB vaccine compared to those with. Those with a history of tooth extraction were 6.90 times (95% CI = 2.66–17.88) more likely to be HBV infected compared to those without. Those who shared sharp equipment were 2.90 times (95% CI = 1.07–7.82) more likely to be HBV infected compared to those who did not share (Table 5).

**Table 5:** Factors associated with HBV infection in multivariable logistic regression analysis

Characteristics	AOR (95%CI)	p-value
**Occupation**		
Employee	1.00	
Business	3.35(1.24–9.08)	0.017*
Healthcare	1.76(0.37–8.26)	0.468
Jobless	3.78(1.17–12.20)	0.026*
**Income USD** $		
<200	5.20 (1.61–16.79)	0.006*
200–400	3.59 (1.38–9.34)	0.009*
>400	1	
**History of STD**		
Yes	4.83 (2.03-11. 50)	< 0.001
No	1	
**History of HB vaccination**		
Yes	1	
No	13.81 (2.46–77.41)	0.003*
**History of Tooth extraction**		
Yes	6.90 (2.66–17.88)	< 0.001*
No	1	
**History of sharing sharp equipment**		
Yes	2.90 (1.07–7.82)	0.036*
No	1	

*Significant level at a p-value < 0.05

## Discussion

Blood donors in Mogadishu, Somalia face a substantial prevalence of hepatitis B virus (HBV) infections, particularly among low-income and unemployed donors, individuals with a history of sexually transmitted diseases (STDs), those with a history of tooth extraction, individuals who engage in the sharing of sharp equipment, and those lacking a history of HB vaccine. Remarkably, only 15.0% of the study participants reported a history of HB vaccine, underscoring the endemic nature of HBV in Somalia [17].

The prevalence reported in this study, even though is high, is equal to a similar study conducted in the Democratic Republic of Congo [34]. However, it falls below the rates observed in comparable studies conducted in Nigeria [9], Jijiga Ethiopia [12], Djibouti [31], Ghana [32], Equatorial Guinea [33], and Burkina Faso [34]. Conversely, this prevalence surpasses that observed in Calabar Nigeria [10], Kenya [11], Eritrea [13], the USA [35], Brazil [36], Mexico [37], Canada [38], Colombia [39], Australia [14], various periods in China [40, 41], India [42], Karachi and Lahore, Pakistan [43, 44], Nepal [45], Thailand [46], Tanzania [47], and Ethiopia [48].

These disparities can be attributed to diverse blood donor risk factors, variations in population and geographical location, discrepancies in vaccine coverage and availability, differential levels of infection exposure, distinct risk factors, individual beliefs, cultural influences, demographic and economic differences, variations in immunity status, and discrepancies in sample sizes.

This study revealed that the likelihood of HBV infection is higher among low-income and jobless blood donors compared to their counterparts. Generally, poverty and infectious diseases are interlinked, particularly HBV infection due to lower socioeconomic status often associated with poorer living habits [49, 50]. For instance, sharing personal items like razors or nail clippers among individuals in poverty may increase the risk of infection, as HBV can survive outside of the body for at least seven days [51]. Furthermore, in the study area, individuals with low income often engage in low-paid jobs, such as waste scavengers using bare hands, which expose them to a higher risk of infection through injuries caused by sharp instruments in the waste including healthcare waste since this study discovered that those who shared sharp equipment were two times more likely to be HBV infected compared to those who did not share. Occupying such high-infection exposure occupations without the use of protective items, coupled with low HB vaccination coverage, poses a significant risk, and may worsen the situation. In addition, financially disadvantaged Somali individuals face challenges in accessing healthcare, as HBV is endemic, and its vaccine is not provided for free in the study area. A similar study supports these findings, indicating that low-income and unemployed individuals would be less willing to pay for the HB vaccine [52]. Moreover, individuals with low income often have a lower level of education, leading to a reduced awareness of HBV infection, which adds to the overall risk. Similar research studies are supported with these observations [51–56].

One notable identified risk factor was that those with a history of STD were four times more likely to be HBV-infected compared to those without. Individuals with a history of STDs may engage in risky behaviors, such as having multiple sexual partners without protection or engaging with high-risk group sex partners, thereby increasing the risk of contracting Hepatitis B [57]. Moreover, individuals infected with STDs may be susceptible to HBV transmission through similar modes, with certain STDs like Syphilis, Herpes, Human Papillomavirus, Chancroid, Gonorrhea, and Chlamydia causing genital sores, warts, or ulcers. These genital lesions create a direct pathway for the HBV virus to enter the bloodstream during sexual contact, posing an elevated risk of HBV transmission. A parallel study also noted that individuals co-infected with STDs and HBV are more likely to transmit HBV through these genital ulcers during sexual contact compared to those without co-infections [58]. Several studies supported these findings [59].

This study highlights that individuals without a history of HB vaccine face a higher risk of HBV infection compared to those who have been vaccinated. Only 15.0% of the study participants had received at least one dose of the HB vaccine, indicating a notably low vaccination coverage compared to the recommended standards. The HB vaccine has proven to be an effective preventive measure, leading to a significant reduction in global HBV epidemiology. Unfortunately, in the study area, limited availability, and high costs of the vaccine act as barriers to achieving national HB vaccination coverage, thereby increasing the risk of infection. A separate study assessing HB vaccination coverage among Somali people found that 2.8%, 16.0%, and 33.4% of healthcare workers and medical students were not fully HB vaccinated and cited vaccine unavailability and high vaccine costs as reasons for not getting vaccinated [60–62]. This study underscores the importance of improving vaccine uptake by addressing issues related to vaccine availability and reducing associated costs.

This study revealed that individuals with a history of tooth extraction or dental repair are at a higher risk of HBV infection compared to those without such a history. This association may be attributed to the potential contamination of dental instruments and inadequate or insufficient sterilization practices. In regions where HBV is endemic, such as the study area, improper infection control measures during tooth procedures can pose a significant risk due to the involvement of blood or other bodily fluids. Implementing effective infection control practices among healthcare workers, which include the sterilization of medical instruments and the use of gloves, masks, and eye protection, is crucial in preventing HBV infections during dental procedures [63]. These practices are strongly recommended in the study area. A study conducted in Ethiopia reached similar conclusions and recommended educational and awareness programs for healthcare workers to discourage traditional dental procedures that may increase the risk of HBV infection [64]. Other studies have also supported these findings [63–68].

## Conclusion

The blood donors in Mogadishu, Somalia, experiencing a high prevalence of Hepatitis B virus (HBV) infection, particularly among individuals with low income and joblessness, those with a history of sexually transmitted diseases (STD), those with a history of tooth extraction, those sharing sharp equipment, and those lacking a history of HB vaccination. Implementation efforts against Hepatitis virus (HBV) infection should specifically focus on low-income individuals, the jobless, and donors with a history of STD to mitigate the burden of HBV infection and promote a safer blood donation environment, ultimately contributing to public health improvement in the region. In addition, discouraging the sharing of sharp equipment, improving infection control practices during tooth extraction procedures, and enhancing HB vaccination uptake, particularly among individuals lacking a history of HB vaccine, is highly recommended.

## Data Availability

All datasets generated and analysed during the current study are included in this article.

## Declarations

### Limitations of the study

While the study exhibits various strengths, it is essential to recognize its limitations. Firstly, the cross-sectional design’s nature hinders the establishment of causal relationships. Furthermore, the study questionnaire wasn’t translated into the Somali language; instead, verbal translation occurred during data collection, as English serves as the common language among educated individuals and officials.

## Abbreviations

HBV: Hepatitis B Virus
CDC: Centers for Disease Control and Prevention
STD: sexual transmission disease
HBsAg: hepatitis B surface antigen
